# Comparison of high and low *trans*-fatty acid consumers: analyses of UK National Diet and Nutrition Surveys before and after product reformulation

**DOI:** 10.1017/S1368980017002877

**Published:** 2017-11-21

**Authors:** Jayne Hutchinson, Holly L Rippin, Jo Jewell, Joao J Breda, Janet E Cade

**Affiliations:** 1 Nutritional Epidemiology Group (NEG), School of Food Science and Nutrition, University of Leeds, Leeds LS2 9JT, UK; 2 Division of Noncommunicable Diseases and Promoting Health through the Life-Course, WHO Regional Office for Europe, Copenhagen, Denmark

**Keywords:** *Trans*-fatty acids (TFA), Industry product reformulation, Artificial TFA reduction, WHO TFA recommendations, Socio-economic disadvantage, Consumer characteristics

## Abstract

**Objective:**

The WHO encourages the virtual elimination of artificial *trans*-fatty acids (TFA), which increase CHD risk. Our UK analysis explores whether voluntary reformulation results in differential TFA intakes among socio-economic groups by determining characteristics of high TFA consumers before and after product reformulation.

**Design:**

Food intake was collected by 7d weighed records pre-reformulation and 4d diaries post-reformulation. Sociodemographic characteristics of TFA consumers above the WHO limit, and of the top 10 % of TFA consumers as a percentage food energy, were compared with those of lower TFA consumers. Multivariate logistic regression determined independent socio-economic predictors of being a top 10 % consumer.

**Subjects:**

UK National Diet and Nutrition Surveys (NDNS) for adults aged 19–64 years pre-reformulation (2000/01; *N* 1724) and post-reformulation (2010/11–2011/12; *N* 848).

**Results:**

Post-reformulation 2·5 % of adults exceeded the WHO limit, *v*. 57 % pre-reformulation. In unadjusted analyses, high TFA consumption was associated with lower income, lower education and long-term illness/disability pre- but not post-reformulation. In adjusted pre-reformulation analyses, degree holders were half as likely as those without qualifications to be top 10 % consumers (OR=0·51; 95 % CI 0·28, 0·92). In adjusted post-reformulation analyses, those with higher income were 2·5–3·3 times more likely to be top 10 % consumers than lowest income households. Pre-reformulation, high consumers consumed more foods containing artificial TFA, whereas ruminant TFA were more prominent post-reformulation.

**Conclusions:**

High TFA consumption was associated with socio-economic disadvantage pre-reformulation, but evidence of this is less clear post-reformulation. Voluntary reformulation appeared effective in reducing TFA content in many UK products with mixed effects on dietary inequalities relating to income and education.

*Trans*-fatty acid (TFA) consumption is associated with increases in all-cause mortality^(^ ^1^ ^)^; for every 2 % of total energy intake (%TE) from TFA, CHD increases by 23 %^(^ ^2^ ^)^. There are two forms of TFA: one occurs naturally through biohydrogenation in the stomach of ruminant animals and the other is produced artificially in processed foods by hydrogenating vegetable or fish oils with hydrogen and a metal catalyst^(^ ^3^ ^)^. Although evidence of increased health risks from industrial *trans*-fatty acids (iTFA) is strong, a systematic review and meta-regression analysis reported that both industrial and ruminant TFA consumption were positively associated with reduced HDL, increased LDL and LDL:HDL cholesterol levels, which are directly associated with increased risk of CVD^(^ ^4^ ^)^.

Common iTFA sources include bakery products, processed meats, fat spreads, savoury snacks and fried fast foods. iTFA were added to processed foods to improve shelf-life, stability and palatability at a lower cost^(^ ^5^ ^)^. TFA removal has long been part of WHO global nutrition guidance^(^ ^6^ ^)^ including the global action plan on prevention and control of non-communicable diseases^(^ ^7^ ^)^. The 2003 WHO/FAO technical report no. 916 stated that TFA intake should be minimised (<1 %TE)^(^ ^8^ ^)^. WHO called for a ‘virtual elimination’ from the food supply, based on evidence that setting legal TFA limits (typically 2 g/100 g total fat) in food products is effective in meeting these intake goals^(^ ^9^ ^)^. iTFA reduction has also been part of UK public health policy since 2011, including the Public Health Responsibility Deal, when the food industry confirmed ongoing efforts to remove iTFA from their products via a formal pledge^(^ ^10^ ^)^. Consequently, pressure on food manufacturers and retailers to reduce or remove iTFA has increased in recent years. Unlike some European countries which have introduced bans (including Austria, Denmark and Hungary)^(^ ^11^ ^–^ ^14^ ^)^, the UK has largely pursued TFA reduction via voluntary product reformulation e.g. by removing partially hydrogenated vegetable oils from foods and using advanced production techniques like modifying the fats and oils used in food preparation^(^ ^10^ ^)^.

Reducing iTFA intake could substantially reduce CHD mortality and health inequalities^(^ ^15^ ^)^. Modelling techniques indicate a 1 % reduction of TFA in daily energy intake could result in 3900 fewer deaths and 37 000 life-years gained, with the most deprived quintile benefitting the most. This modelling accounted for CHD mortality disproportionately affecting lower socio-economic groups^(^ ^16^ ^)^, but assumed equal TFA consumption. However, this reduction in deaths for the most deprived is likely to be underestimated because TFA may have been higher in this group^(^ ^17^ ^)^. The UK Low Income Diet and Nutrition Survey (LIDNS) 2003–2005 reported higher adult TFA intakes than the earlier 2000/01 UK nationally representative survey, both of which used pre-reformulation TFA values^(^ ^17^ ^)^. The National Diet and Nutrition Survey (NDNS) data collected in 2008–2012^(^ ^18^ ^)^ show that average UK TFA intake meets the UK Dietary Reference Value of <2 % of food energy (%FE) from TFA^(^ ^19^ ^)^ and the WHO recommended limit of <1 %TE^(^ ^8^ ^)^. However, these averages potentially mask problems associated with higher intakes in certain groups^(^ ^20^ ^)^.

Previous reviews have suggested that, globally, voluntary measures may be less effective than legislation in reducing TFA and intake inequalities^(^ ^21^ ^)^. In part, this may be due to difficulties ensuring the participation of a critical mass of manufacturers and retailers, especially small and medium-sized enterprises, which dominate the food sector^(^ ^22^ ^)^. In New York State some counties imposed iTFA limits of 0·5 g/portion in food-service establishments, resulting in an estimated 4·5 % reduction in CVD-related deaths^(^ ^23^ ^)^. Elsewhere, a ban has been favoured to maximise impact for all socio-economic groups and create a level playing field for companies. For example, CVD mortality in Denmark fell more than expected following a ban on TFA above 2 g/100 g fats/oils in 2003^(^ ^24^ ^)^. The UK experience with TFA reformulation is therefore a good case study to explore the potential impact, strengths and limitations of voluntary reformulation^(^ ^22^ ^)^.

Our research analysed pre-reformulation and post-reformulation TFA data from the UK NDNS to determine characteristics of high TFA consumers compared with lower consumers at these different time points, highlighting the potential impact of reformulation with particular reference to socially disadvantaged groups.

## Methods

NDNS dietary and sociodemographic data were analysed and compared from surveys representing before (2000/01) and after (2010–2012) TFA product reformulation. The pre-reformulation analysis used NDNS data for 1724 UK adults aged 19–64 years collected in 2000/01^(^ ^25^ ^)^. The separate post-reformulation analysis used data which incorporated the reduced TFA content of reformulated products for 848 adults (restricted to ages 19–64 years) from Years 3 and 4 (2010/11 and 2011/12) of the 2008–2012 NDNS Rolling Programme (RP)^(^ ^18^ ^)^. Years 1 and 2 of the NDNS RP were not included in the analyses because NDNS RP Year 1 data did not incorporate post-reformulation TFA compositions and Year 2 data incorporated only some changes. The samples were drawn from the GB and UK Postcode Address Files, selected using multistage random probability sampling with postal sectors as the primary sampling units. All food and nutrient variables were derived from short-term food records or diaries and sociodemographic variables were derived from NDNS questionnaire responses collected alongside food intake.

The NDNS 2000/01 collected food data using 7d weighed intake dietary records for all foods and drinks consumed^(^ ^25^ ^)^. The NDNS 2008–2012 RP used a 4d consecutive food diary and portion sizes were estimated using household measures and food packaging labels^(^ ^18^ ^)^. TFA values in the composition databanks underpinning the NDNS 2000/01 survey were based mainly on food composition analyses carried out in the 1990s, using composite samples of various brands of similar foods. When no analytical data for a food were available, the TFA value was estimated using manufacturer/retailer data for total and saturated fat (typically from the product label) and the fatty acid profile of similar foods^(^ ^26^ ^)^. Updates on laboratory-analysed TFA levels in processed foods high in iTFA which had been targeted for reformulation were reported in 2011 and 2013^(^ ^27^ ^,^ ^28^ ^)^. These included biscuits, buns, cakes, pastries and products bought in 2008 and reported in 2011^(^ ^27^ ^)^. The 2013 report included pizza, garlic bread, breakfast cereal, quiche, fat spreads, cooking fats and oils, chicken products, meat pies, fish products, chips, coleslaw, crisps and savoury snacks, confectionary, chocolate spread, soup, baby rusks and ice cream^(^ ^28^ ^)^. The UK Department of Health incorporated these values into the nutrient databanks supporting Years 3 and 4 (2010/11 and 2011/12) of the NDNS RP. Forty-three per cent of the 2900 products (which included naturally occurring TFA) in the Year 1 nutrient databanks, with non-zero TFA values in Year 1 and Year 4, had updated TFA values in the Year 4 nutrient databank. Only the main product groups for milk, fruit, salads and raw vegetables, drinks and supplements did not have TFA value updates. The products analysed for the update were purchased between 2008 and 2010 and were mostly popular and widely purchased products in the UK^(^ ^27^ ^,^ ^28^ ^)^. Sub-samples of food products had been combined in equal weights to form a composite sample for analysis, with five to sixteen sub-samples for each category^(^ ^28^ ^)^. The UK Department of Health incorporated the new TFA values into the new composition tables.

### Statistical methods

Two methods of grouping NDNS individuals were used to compare the characteristics of high and lower TFA consumers:

1.
individuals who consumed over the current WHO recommended limit on TFA^(^ ^8^ ^)^, i.e. ≥1 %TE from TFA, compared with those who consumed <1 %TE from TFA; and
2.
the top 10 % of TFA consumers in terms of percentage of energy from food intake compared with the remaining 90 %.

The second analysis was undertaken because it provided more power to find associations between high TFA consumption and sociodemographic variables. It also excludes alcohol from energy intake, which may dilute findings. For each analysis, the following sociodemographic characteristics were compared for adults: (i) age, continuous and grouped (19–34, 35–49, 50–64 years); (ii) gender; (iii) qualifications (no qualifications; school certificates and other qualifications; higher education below degree; degree); (iv) in employment (i.e. economically active or in full-time education, yes/no); (v) gross income (split into five groups); (vi) social class (NDNS 2000/01 used the Registrar General’s Standard Occupational Classification and NDNS 2010–2012 used the National Statistics Socio-economic Classification); (vii) region (Northern England; Midlands; London, East and South England; Scotland, Wales (and Northern Ireland for NDNS RP Years 3 and 4)); (viii) number of adults in household (aged over 16 years); (ix) child in household (yes/no); (x) marital status (single/never married; married and living together/cohabiting; separated/divorced/widowed); (xi) White or non-White; (xii) ethnic group (White, Black or Black British, Asian or Asian British, Other group); and (xiii) longstanding illness or disability (yes/no). NDNS income data were provided in income bands and not as a continuous variable; we collapsed these into five bands (see notes to tables) so that the nationally representative weighted percentages in the respective bands were similar in both surveys. Additionally, for the NDNS 2010–2012 analyses the following comparisons were undertaken: (xiv) Index of Multiple Deprivation (IMD) by quintile; and (xv) equivalised household income by continuous variable and quintile. Potential associations between high TFA and alcohol intake were assessed using alcohol intake data on the day of highest alcohol consumption in the seven days (none, within daily recommended levels, between recommended and binge drinking levels, binge drinking levels; results not shown in tables).

In these univariate analyses, the means of continuous data were compared using *t* tests and categorical data were compared using *χ* ^2^ tests. Mean intakes for selected nutrients that were reasonably normally distributed were compared by *t* tests between the high and lower TFA groups. Macronutrients: TFA (g, %TE, %FE); total energy (kJ/kcal); food energy (kJ/kcal); fat (%FE); saturated fat (%FE); non-milk extrinsic sugars (%FE). Micronutrients per 4184 kJ (1000 kcal): Na (mg); vitamin C (mg); vitamin D (µg); vitamin E (mg). Additionally, the individual percentage contribution of all main food and beverage groups (approximately sixty) to total food energy intake and total TFA intake were analysed; those contributing less than 0·5 % were not tabled.

Multivariate logistic regression analyses of the NDNS data sets were undertaken to determine which sociodemographic characteristics were independently associated with high TFA consumption over other characteristics. Due to low numbers in the post-reformulation surveys consuming above the WHO recommended TFA limit and the potential distortion of total energy intake by high alcohol consumers, multivariate analyses were conducted on the top 10 % of TFA consumers as a percentage of food energy intake (rather than total energy intake). For the multivariate regression analyses, we included the following variables common to both the pre- and post-reformulation NDNS surveys: food energy intake per 418 kJ (100 kcal); age (continuous); gender; number of adults in household (continuous); number of children in household (continuous); and (as categorised above): qualification, income, region, marital status, ethnicity and longstanding illness. The variable for IMD quintiles was included in sensitivity analyses for the recent NDNS RP; however, this information was not available for analyses on the earlier NDNS 2000/01 data set.

Within- and between-person variations in TFA intake were calculated to produce a predicted ‘usual’ intake distribution and to re-estimate the proportion above the WHO recommended limit for both pre- and post-reformulation surveys. The common framework method with square-root-transformed intakes was used, as described in Dodd *et al*.^(^ ^29^ ^)^. Because no individual in the recent NDNS (2010–2012) survey had TFA intake over the WHO recommendation using usual intake calculations, it was not possible to create new TFA consumer groups with this method and repeat the comparative analyses described above. Additionally, because the usual intake model shrinks all intakes towards the mean, the same individuals would be in the top 10 % of the usual intake distribution as found in the original top 10 % of TFA consumers; therefore, additional tables for this could not be produced.

The response rate for completing a 7d diary in the NDNS 2000/01 was 47 % of the eligible sample; and for the NDNS RP was 53 % for Year 3 and 55 % for Year 4 recording dietary intake. Analyses were weighted using the NDNS survey weights provided, to produce estimated results representative of the UK population, accounting for non-responses^(^ ^30^ ^,^ ^31^ ^)^. Statistical significance was set at *P*<0·05. Analyses used the Stata statistical software package versions 13 and 14.

## Results

The age ranges in the nationally representative weighted pre- and post-reformulation samples were similar, being 37, 33 and 30 % for the age groups of 19–34, 35–49 and 50–64 years, respectively, in the UK NDNS 2000/01, and 35, 36 and 29 %, respectively, in Years 3 and 4 (2010/11 and 2011/12) of the recent UK NDNS RP. The proportion of males (49 and 48 %) was also similar. There were differences regarding ethnicity and degree attainment: 94 % were White and 19 % held a degree in the pre-reformulation surveys, whereas 85 % were White and 28 % held a degree in the post-reformulation surveys.

In the UK NDNS 2000/01, on average men and women aged 19–64 years consumed 1·1 % of their total and 1·2 % of their food energy from TFA. In these pre-reformulation analyses, 57 % of adults (57 % of males; 58 % of females) consumed over the current WHO recommended TFA limit, i.e. ≥1 %TE from TFA. The average consumption of those consuming over the WHO limit was 1·4 % of total and food energy intake. In terms of the UK TFA recommendations, only 4 % of men and women consumed ≥2 %FE from TFA.

In Years 3 and 4 (2010/11 and 2011/12) of the recent UK NDNS RP, on average adults aged 19–64 years consumed 0·51 % of their total (0·50 % for men; 0·52 % for women) and 0·53 % of their food energy (0·53 % for men; 0·54 % for women) from TFA. On average, 2·5 % of adults consumed over the current WHO TFA limit (1·9 % of males; 3·0 % of females). The average TFA intake of those consuming above the WHO limit was 1·2 %TE and 1·3 %FE. In terms of the UK TFA recommendation, only one woman (<0·01 %) and no men (0 %) aged 19–64 years in this survey consumed ≥2 %FE from TFA.

### Sociodemographic characteristics of higher consumers of *trans*-fatty acids

In the UK NDNS 2000/01 unadjusted analyses, adults consuming over the WHO recommended TFA limit were significantly more likely to have middle to lower incomes, no qualifications and be White than those below the WHO TFA limit (Table 1). No significant differences were found regarding other sociodemographic data. Conversely, in the recent UK NDNS (2010/11 and 2011/12), individuals consuming over the WHO TFA limit did not have significantly different sociodemographic characteristics compared with those who consumed less (Table 1). Although a higher proportion of White individuals and those with no qualifications consumed over the WHO TFA recommendation post-reformulation, the difference was not significant. In relation to alcohol intake, individuals consuming below the WHO TFA limit were more likely to be binge drinkers than those consuming TFA above recommendations pre-reformulation (38·5 *v.* 24·6 %, *P*<0·001), but not post-reformulation.

**Table 1:** Sociodemographic characteristics of adults (aged 19–64 years) in the National Diet and Nutrition Survey (NDNS 2000/01) and in Years 3 and 4 of the National Diet and Nutrition Survey Rolling Programme (NDNS RP 2010/11 and 2011/12) who consumed 1 % or more of their total energy intake from *trans*-fatty acids (TFA) compared with those who consumed less

	NDNS 2000/01				Years 3 & 4 of NDNS RP 2010/11 and 2011/12			
	Total (*N* 1724, weighted *N* 1724)	≥1 %TE from TFA (*N* 988, weighted *N* 988)	<1 %TE from TFA (*N* 736, weighted *N* 736)		Total (*N* 848, weighted *N* 1277)	≥1 %TE from TFA (*N* 22, weighted *N* 32)	<1 %TE from TFA (*N* 826, weighted *N* 1245)	
Adults: WHO TFA limit	Unweighted *n*	%*	%*	*P* value	Unweighted *n*	%*	%*	*P* value
TFA (g), mean		3·11	1·59	<0·001		2·18	1·02	<0·001
sd		1·30	0·66			0·62	0·53	
Age (years), mean		40·96	40·04	0·2		40·94	41·30	0·9
sd		12·85	12·50			14·83	12·79	
Age group				0·6				0·9
19–34 years	510	36·8	37·9		245	33·9	34·6	
35–49 years	682	32·6	33·7		330	33·3	36·3	
50–64 years	532	30·6	28·4		273	32·8	29·1	
Males	766	48·0	48·8	0·8	484	38·5	49·5	0·4
Qualifications				0·03				0·5
No qualifications (or in full-time education)	301	18·1	13·3		138	18·2	16·7	
School certificates & other qualifications	856	51·3	51·3		374	51·2	43·9	
Higher education below degree	245	13·3	14·6		97	0·00	11·2	
Degree	321	17·2	20·8		237	30·5	28·2	
In employment	1280	77·8	75·8	0·3	609	82·5	73·1	0·3
Gross household income†				<0·001				0·7
Lowest income group	243	10·6	11·0		123	14·2	12·9	
2	267	15·0	10·3		141	10·5	19·1	
3	253	17·4	11·8		85	18·1	10·3	
4	381	24·1	24·5		141	15·9	20·3	
Highest income group	548	32·9	42·4		239	41·3	37·4	
Equalised household income (£), mean	NA	NA	NA		729	37 798	32 874	0·5
sd						27 512	23 885	
Equivalised household income								0·9
Lowest income quintile	NA	NA	NA		129	14·2	17·3	
2					114	13·6	16·4	
3					124	11·5	19·1	
4					170	29·0	22·0	
Highest income quintile					192	31·6	25·2	
Quintile of IMD score, England only								0·7
Most deprived	NA	NA	NA		131	9·8	15·5	
2					142	9·2	18·4	
3					157	21·6	18·7	
4					138	14·0	16·8	
Least deprived					133	22·3	15·8	
Other UK countries, not England					147	23·1	15·5	
Socio-economic classification‡				0·8				0·9
Highest	100	6·5	5·4		361	46·2	44·4	
	528	27·7	33·8		170	16·9	20·5	
	401	23·5	25·3		300	36·9	35·2	
	282	18·8	16·1					
	283	17·0	14·7					
Lowest	98	6·5	4·8					
Region				0·6				0·7
Northern England	451	25·8	28·3		201	28·8	23·6	
Midlands	266	16·3	14·3		148	16·3	15·5	
London, East & South England	808	46·0	45·0		352	32·5	45·1	
Scotland, Wales (+NI in NDNS RP)	199	11·9	12·3		147	22·3	15·8	
Number of adults in household, mean		2·28	2·22	0·3		2·37	2·30	0·8
sd		0·93	0·90			0·98	0·94	
Child in household, yes	644	36·1	38·0	0·4	328	49·8	39·4	0·4
Marital status				0·8				0·2
Single/never married	358	19·7	21·1		188	34·8	20·1	
Married/living together/cohabiting	1075	70·6	69·0		530	54·2	70·9	
Separated/divorced/widowed	291	9·8	9·9		130	11·0	9·0	
Ethnic group, two categories				<0·001				0·2
White	1629	95·6	91·7		750	93·1	85·0	
Non-White	95	4·4	8·3		98	6·9	15·0	
Ethnic group				0·001				0·8
White	1629	95·6	91·7		750	93·1	85·0	
Black or Black British	29	0·9	2·5		29	4·1	4·0	
Asian or Asian British	35	2·1	3·2		40	2·9	6·5	
Any Other group incl. mixed	31	1·4	2·6		29	0·0	4·5	
Has longstanding illness, yes	655	37·9	34·9	0·3	292	30·7	31·3	1·0

%TE, percentage of total energy; NI, Northern Ireland; NA, not available; TFA intake ≥1%TE is above the WHO recommended TFA limit.

* Values presented are percentages, unless indicated otherwise.†Gross annual household income in the previous 12 months groupings for NDNS 2000/01 are: <£8000, £8000 to <£12 000, £12 000 to <£18 000, £18 000 to <£25 000, ≥£25 000; for NDNS RP are: <£15 000, £15 000 to <£20 000, £20 000 to <£30 000, £30 000 to <£40 000, ≥£40 000.

‡ Social class groups for NDNS 2000/01 are: I, professional; II, managerial and technical; IIIN, skilled non-manual; IIIM, skilled manual; IV, semi-skilled; V, unskilled; socio-economic classification grouping for NDNS RP are: Managerial & Professional, Intermediate & Small businesses, Routine & Never worked.

In the NDNS 2000/01 unadjusted analyses, some sociodemographic characteristics were associated with being in the top 10 % of TFA consumers in terms of percentage of food energy (Table 2). As found for those consuming above the WHO recommended TFA limit, the top 10 % consumers were more likely to have middle to lower incomes, no qualifications and longstanding illnesses. Regional differences were also found, but there were no differences regarding ethnicity. In the recent NDNS RP (2010/11 and 2011/12), in contrast to the NDNS 2000/01, income was not associated with being in the top 10 % of TFA consumers (Table 2). The top 10 % of TFA consumers were more likely than the remaining 90 % to have no qualifications and reside in the most deprived areas of England.

**Table 2:** Sociodemographic characteristics of adults (aged 19–64 years) in the National Diet and Nutrition Survey (NDNS 2000/01) and Years 3 and 4 of the National Diet and Nutrition Survey Rolling Programme (NDNS RP 2010/11 and 2011/12) who were the top 10 % of *trans*-fatty acid (TFA) consumers as a percentage of food energy compared with the remaining 90 %

	NDNS 2000/01				NDNS RP 2010/11 and 2011/12			
Adults: top 10 % TFA	Total (*N* 1724, weighted *N* 1724)	Top 10 % TFA consumers as %FE (*N* 191, weighted *N* 176)	Remaining 90 % (*N* 1533, weighted *N* 1548)		Total (*N* 848, weighted *N* 1277)	Top 10 % TFA consumers as %FE (*N* 88, weighted *N* 130)	Remaining 90 %(*N* 760, weighted *N* 1147)	
consumers	Unweighted *n*	%*	%*	*P* value	Unweighted *n*	%*	%*	*P* value
TFA intake (g), mean		4·55	2·22	<0·001		1·93	0·95	<0·001
sd		1·00	1·85			0·59	0·46	
Age (years), mean		41·94	40·41	0·2		42·90	41·10	0·3
sd		12·95	12·68			13·12	12·80	
Age group				0·3				0·5
19–34 years	510	31·7	37·9		245	29·0	35·2	
35–49 years	682	33·9	33·0		330	36·9	36·2	
50–64 years	532	34·4	29·1		273	34·1	28·6	
Male	766	50·5	48·1	0·5	484	55·4	48·6	0·3
Qualifications				0·03				0·2
No qualifications (or in full-time education)	301	23·1	15·1		138	23·6	15·9	
School certificates & other qualifications	856	50·4	51·4		374	38·1	44·8	
Higher education below degree	245	14·0	13·9		97	6·2	11·5	
Degree	321	12·4	19·5		237	32·1	27·8	
In employment	1280	74·7	77·2	0·5	609	70·2	73·7	0·5
Gross household income†				0·03				0·6
Lowest income group	243	12·7	10·6		123	8·8	13·4	
2	267	15·3	12·7		141	23·3	18·4	
3	253	22·2	14·2		85	14·2	10·0	
4	381	20·4	24·7		141	19·7	20·2	
Highest income group	548	29·5	37·8		239	33·9	38·0	
Equalised household income (£), mean	NA	NA	NA		729	33 040	32 989	1·0
sd						23 396	24 057	
Equivalised household income								0·8
Lowest income quintile	NA	NA	NA		129	13·8	17·6	
2					114	21·4	15·7	
3					124	17·8	19·1	
4					170	21·5	22·3	
Highest income quintile					192	25·5	25·3	
Quintile of IMD score, England only								0·03
Most deprived	NA	NA	NA		131	27·4	14·4	
2					142	8·7	19·2	
3					157	15·5	18·8	
4					138	18·3	16·7	
Least deprived					133	17·2	14·6	
Other UK countries, not England					147	13·0	16·3	
Socio-economic classification‡				0·2				0·6
Highest	100	6·6	6·0		361	43·7	44·5	
	528	21·7	31·2		170	16·6	20·8	
	401	22·9	24·4		300	39·8	34·7	
	282	19·5	17·4					
	283	19·5	15·6					
Lowest	98	9·8	5·3					
Region				0·006				0·2
Northern England	451	23·3	27·3		201	23·8	23·8	
Midlands	266	13·4	15·7		148	24·2	14·5	
London, East & South England	808	56·6	44·4		352	39·0	45·5	
Scotland, Wales (+NI in NDNS RP)	199	6·7	12·7		147	13·0	16·3	
Number of adults in household, mean		2·15	2·27	0·2		2·13	2·32	0·07
sd		0·90	0·92			0·77	0·95	
Child in household, yes	644	37·3	36·9	0·9	328	39·6	40·1	0·9
Marital status				0·06				0·4
Single/never married	358	17·7	20·6		188	24·8	20·0	
Married & living together/cohabiting	1075	67·3	70·2		530	64·2	71·2	
Separated/divorced/widowed	291	15·0	9·2		130	11·0	8·8	
Ethnic group, two categories				0·5				0·4
White	1629	95·6	93·8		750	81·5	85·6	
Non-White	95	4·4	6·2		98	18·5	14·4	
Ethnic group				0·6				0·7
White	1629	95·6	93·8		750	81·5	85·6	
Black or Black British	29	0·5	1·7		29	6·6	3·7	
Asian or Asian British	35	2·6	2·6		40	6·9	6·4	
Any Other group incl. mixed	31	1·3	2·0		29	5·0	4·4	
Has longstanding illness, yes	655	46·7	35·5	0·01	292	38·0	30·5	0·2

%FE, percentage of food energy; NI, Northern Ireland; NA, not available.

* Values presented are percentages, unless indicated otherwise.†Gross annual household income in the previous 12 months groupings for NDNS 2000/01 are: <£8000, £8000 to <£12 000, £12 000 to <£18 000, £18 000 to <£25 000, ≥£25 000; for NDNS RP are: <£15 000, £15 000 to <£20 000, £20 000 to <£30 000, £30 000 to <£40 000, ≥£40 000.

‡ Social class groups for NDNS 2000/01 are: I, professional; II, managerial and technical; IIIN, skilled non-manual; IIIM, skilled manual; IV, semi-skilled; V, unskilled; socio-economic classification grouping for NDNS RP are: Managerial & Professional, Intermediate & Small businesses, Routine & Never worked.

In the multivariate logistic regression analyses of the top 10 % of adult TFA consumers in the pre-reformulation NDNS 2000/01, there were significant differences relating to education and region (Table 3). Adults with a degree were half as likely to be top 10 % TFA consumers as those with no qualifications (adjusted OR=0·51; 95 % CI 0·28, 0·92; Table 3). Individuals living in the North of England (adjusted OR=0·53; 95 % CI 0·33, 0·85), Scotland and Wales (adjusted OR=0·33; 95 % CI 0·19, 0·60) were less likely to be in the top 10 % of TFA consumers than adults in living in London, East and South of England. However, unlike the unadjusted analyses, there was no evidence of significant differences relating to income and long-term illness in the multivariate analyses. In contrast, multivariate logistic regression analyses of the recent NDNS RP showed income-related differences as significant. Higher income groups were 2·5–3·25 times more likely to be top 10 % TFA consumers than those in the lowest income group; those with household income of £20 00–30 000 were most likely to be high TFA consumers than households with income below £15 000 (adjusted OR=3·25; 95 % CI 1·10, 9·62). Regional differences also became significant in multivariate analyses of the NDNS RP, but unlike in the earlier NDNS 2000/01, the top 10 % of consumers were more likely to reside in the Midlands than London, East and South England (adjusted OR=2·40; 95 %CI 1·13, 5·11). In sensitivity analyses, inclusion of the IMD variable quintiles into the NDNS RP multivariate analyses did not change most of the results substantially. However, being a top 10 % TFA consumer was about 70 % less likely for those with higher education below degree compared with those with no education (adjusted OR=0·25; 95 % CI 0·07, 0·97) and for those in the second most deprived group compared with the most deprived (adjusted OR=0·27; 95 % CI 0·09, 0·82).

**Table 3:** Odds of being in the top 10 % of *trans*-fatty acid (TFA) consumers as a percentage of food energy compared with the remaining 90 % depending on sociodemographic characteristics of adults (aged 19–64 years) in the National Diet and Nutrition Survey (NDNS 2000/01) and Years 3 and 4 of the National Diet and Nutrition Survey Rolling Programme (NDNS RP 2010/11 and 2011/12)

	Adjusted OR and 95 % CI of being in the top 10 % TFA consumers as %FE					
	NDNS 2000/01 (*N* 1689, weighted *N* 1680)			NDNS RP 2010/11 and 2011/12 (*N* 728, weighted *N* 1068)		
Adult: multivariate analyses top 10 % TFA consumers	OR	95 % CI	*P* value	OR	95 % CI	*P* value
Food energy intake per 418 kJ (100 kcal)	1·01	1·01, 1·01	<0·001	1·00	0·95, 1·06	0·9
Age (per year)	1·00	0·99, 1·02	0·9	1·02	0·98, 1·05	0·3
Gender						
Male	1·00	Ref.	–	1·00	Ref.	–
Female	0·76	0·50, 1·16	0·2	0·85	0·47, 1·55	0·6
Qualification						
No qualifications (or in full-time education)	1·00	Ref.	0·1†	1·00	Ref.	0·2†
School certificates & other qualifications	0·74	0·48, 1·14	0·3	0·43	0·18, 1·04	0·1
Higher education below degree	0·74	0·40, 1·35	0·2	0·27	0·07, 1·03	0·3
Degree	0·51	0·28, 0·92	0·03	0·61	0·23, 1·60	0·3
Gross household income*						
Lowest income group	1·00	Ref.	0·06†	1·00	Ref.	0·05†
2	0·88	0·47, 1·64	0·7	2·78	1·23, 6·22	0·01
3	1·16	0·65 2·09	0·6	3·25	1·10, 9·62	0·03
4	0·57	0·31, 1·05	0·07	2·49	1·08, 5·75	0·03
Highest income group	0·60	0·31, 1·17	0·1	2·57	1·08, 6·14	0·03
Region						
London, East & South England	1·00	Ref.	–	1·00	Ref.	–
North England	0·53	0·33, 0·85	0·008	1·39	0·66, 2·94	0·4
Midlands	0·65	0·39, 1·07	0·09	2·40	1·13, 5·11	0·02
Scotland, Wales (+NI in NDNS RP)	0·33	0·19, 0·57	<0·001	1·19	0·48, 2·92	0·7
Ethnic group						
White	1·00	Ref.	–	1·00	Ref.	–
Black or Black British	0·24	0·03, 2·15	0·2	1·22	0·24, 6·23	0·8
Asian or Asian British	0·84	0·26, 2·73	0·8	1·54	0·44, 5·42	0·5
Other incl. mixed	0·74	0·20, 2·77	0·7	1·34	0·30, 6·04	0·7
Marital status						
Single/never married	1·00	Ref.	–	1·00	Ref.	–
Married & living together/cohabiting	1·00	0·57, 1·75	1·0	0·56	0·21, 1·48	0·2
Separated/divorced/widowed	1·49	0·85, 2·64	0·2	0·71	0·25, 2·02	0·5
Long-term illness or disability						
Yes	1·00	Ref.	–	1·00	Ref.	–
No	0·70	0·49, 1·01	0·06	0·71	0·41, 1·23	0·2
Number of adults in household	0·96	0·78, 1·23	0·7	0·83	0·57, 1·20	0·3
Number of children in household	1·06	0·89, 1·26	0·5	1·07	0·81, 1·39	0·6

%FE, percentage of food energy; NI, Northern Ireland; Ref., reference category.

* Gross annual household income in the previous 12 months groupings for NDNS 2000/01 are: <£8000, £8000 to <£12 000, £12 000 to <£18 000, £18 000 to <£25 000, ≥£25 000; for NDNS RP are: <£15 000, £15 000 to <£20 000, £20 000 to <£30 000, £30 000 to <£40 000, ≥£40 000.

† Test for trend across the groups.

### Foods contributing most to trans-fatty acid intake in higher trans-fatty acid consumers

Pre-reformulation, 76 % of TFA intake in those over the WHO limit was spread across meat (20 %), dairy (20 %), biscuits, buns, cakes, pastries, fruit pies and puddings (21 %) and spreads (15 %; Table 4). For those meeting the recommendation, the largest percentage (50 %) came from meat and dairy. In contrast, post-reformulation, 78 % of TFA intake of those over the WHO limit came from dairy (46 %) and meat (32 %), with cheese, butter, cream, lamb, burgers and kebabs being the highest contributors totalling 62 %. Only 12 % was contributed by biscuits, buns, cakes, pastries, fruit pies and puddings and <1 % by spreads (Table 4). For those meeting the WHO recommendation, smaller proportions of TFA intake came from dairy products (41 %) and meat (26 %), with only 6 % from biscuits, buns, cakes, pastries, fruit pies and puddings.

**Table 4:** Percentage contribution of main food groups to energy intake and average daily *trans*-fatty acid (TFA) intake of adults (aged 19–64 years) in the National Diet and Nutrition Survey (NDNS 2000/01) and Years 3 and 4 of the National Diet and Nutrition Survey Rolling Programme (NDNS RP 2010/11 and 2011/12) who consumed 1 % or more of their total energy intake from TFA compared with those who consumed less

	NDNS 2000/01				NDNS RP 2010/11 and 2011/12			
Adults: WHO TFA limit	Total (*N* 1724, weighted *N* 1724)	≥1 %TE from TFA (*N* 988, weighted *N* 988)	<1 %TE from TFA (*N* 736, weighted *N* 736)	*P* value for ∆	Total (*N* 848, weighted *N* 1277)	≥1 %TE from TFA (*N* 22, weighted *N* 32)	<1 %TE from TFA (*N* 826, weighted *N* 1245)	*P* value for ∆
Main food group name	%FE	%TFA	%TFA	in %TFA	%FE	%TFA	%TFA	in %TFA
Pasta, rice and other cereals	6·3	2·8	3·5	0·06	8·9	0·3	3·4	<0·001
White bread	8·9	0·9	1·4	0·05	7·7	0·8	3·0	<0·001
Wholemeal bread	2·0	0·2	0·5	<0·001	2·6	0·4	1·1	0·001
Brown, granary and wheatgerm					2·5	0·3	0·6	0·004
Other bread	2·7	0·8	1·2	<0·001	0·2	0·0	0·1	<0·001
High-fibre breakfast cereals	3·0	1·1	0·8	0·2	2·2	0·0	0·2	<0·001
Biscuits	2·8	8·6	5·5	<0·001	3·4	0·3	1·1	<0·001
Buns, cakes, pastries & fruit pies	3·8	9·9	6·7	<0·001	3·3	6·1	4·1	0·5
Puddings	1·1	2·0	1·2	<0·001	0·8	6·0	1·5	0·2
Whole milk	1·7	1·3	1·8	0·03	0·9	1·0	1·7	0·2
Semi-skimmed milk	3·1	3·2	5·5	<0·001	2·6	3·5	7·8	<0·001
Other milk and cream	0·6	1·0	0·9	0·2	0·9	10·3	2·4	0·05
Cheese	3·0	7·2	9·6	<0·001	3·4	17·8	17·8	1·0
Yoghurt, fromage frais and dairy	1·2	0·6	1·1	<0·001	1·4	1·3	1·9	0·3
Eggs and egg dishes	2·2	2·4	3·3	0·002	2·0	0·8	2·1	<0·001
Butter	1·3	4·0	3·9	0·9	1·4	11·1	7·1	0·2
PUFA margarine & oils	0·1	0·5	0·4	0·9	0·2	0·0	0·0	
Low-fat spread	0·4	1·6	1·9	0·5	0·3	0·0	0·2	<0·001
Other margarine fats and oils	0·6	4·5	3·3	0·02	0·8	0·1	0·1	0·9
Reduced-fat spread	1·8	8·7	4·1	<0·001	2·0	0·2	1·5	<0·001
Bacon and ham	1·6	0·3	0·6	<0·001	1·6	0·2	1·0	<0·001
Beef, veal and dishes	2·9	3·7	6·3	<0·001	2·5	3·2	8·1	<0·001
Lamb and dishes	0·8	2·5	2·9	0·4	0·8	14·3	4·2	0·05
Pork and dishes	0·9	0·2	0·4	0·001	1·0	0·4	0·6	0·3
Coated chicken	1·0	1·1	1·4	0·1	0·9	0·0	0·7	<0·001
Chicken and turkey dishes	3·9	1·2	3·0	<0·001	4·3	2·1	4·0	0·03
Burgers and kebabs	1·2	2·3	2·6	0·4	1·0	8·5	2·7	0·4
Sausages	1·4	0·5	0·9	<0·001	1·7	0·8	2·1	<0·001
Meat pies and pastries	2·3	7·6	3·9	<0·001	1·8	0·5	1·7	0·001
Other meat and meat products	0·7	0·7	1·1	0·02	0·5	1·8	1·0	0·6
White fish coated or fried	1·2	2·0	2·1	0·6	1·2	0·9	1·8	0·2
Other white fish, shellfish	0·6	0·4	0·6	0·04	0·9	0·1	0·3	0·06
Oily fish	1·2	0·3	0·5	0·06	1·0	0·0	0·2	0·01
Vegetables not raw	3·8	1·3	1·7	0·09	4·0	0·9	1·5	0·3
Chips fried & roast potatoes	5·3	4·4	4·2	0·7	4·4	1·2	2·7	0·08
Other potatoes, potato salads	3·0	0·9	1·0	0·3	2·7	0·2	0·4	0·1
Crisps and savoury snacks	2·0	1·3	1·3	0·9	2·1	0·0	0·5	<0·001
Chocolate confectionery	2·4	3·5	2·8	0·06	2·1	0·3	2·2	<0·001
Miscellaneous	3·0	2·6	3·5	<0·001	3·9	3·0	3·8	0·7
Ice cream	0·6	1·3	1·4	0·7	0·6	0·6	1·4	0·05

%TE, percentage of total energy; %FE, percentage of food energy; %TFA, percentage of TFA intake; TFA intake ≥1%TE is above the WHO recommended TFA limit. Percentage contributions of TFA from food types are weighted means of the individuals’ percentage contributions. Food groups contributing less than 0·5 % TFA to food energy in all TFA groups were not tabled.

Regarding the top 10 % of TFA consumers pre-reformulation, 20 % of TFA came from meat, 14 % from dairy, 23 % from spreads and 24 % from biscuits, buns, cakes, pastries, fruit pies and puddings (Table 5). Biscuits, reduced-fat spreads and meat pies and pasties contributed 38 % of TFA intake, which was significantly higher than in the remaining 90 %. In contrast, post-reformulation, 70 % of TFA for the top 10 % of TFA consumers came from just dairy (46 %) and meat (24 %), and only 8 % from biscuits, buns, cakes, pasties, fruit pies and puddings, and 1 % from spreads (other than butter); similar percentages were found for the remaining 90 % of lower TFA consumers (Table 5). Of note, TFA intakes from butter, cream and lamb were significantly higher for the top 10 % of TFA consumers, and TFA intakes from semi-skimmed milk and beef significantly lower (Table 5). Additionally, the percentage of energy from food groups appeared to have a similar distribution in the pre- and post-reformulation surveys (Tables 4 and 5).

**Table 5:** Percentage contribution of main food groups to energy intake and average daily *trans*-fatty acid (TFA) intake of adults (aged 19–64 years) in the National Diet and Nutrition Survey (NDNS 2000/01) and Years 3 and 4 of the National Diet and Nutrition Survey Rolling Programme (NDNS RP 2010/11 and 2011/12) who were the top 10 % of TFA consumers as a percentage of food energy compared with the remaining 90 %

	NDNS 2000/01				NDNS RP 2010/11 and 2011/12			
Adults: top 10 % TFA consumers Main food group	Total (*N* 1724, weighted *N* 1724)	Top 10 % TFA consumers as %FE (*N* 191, weighted *N* 176)	Remaining 90 % (*N* 1533, weighted *N* 1548)	*P* value for ∆	Total (*N* 848, weighted *N* 1277)	Top 10 % TFA consumers as %FE (*N* 88, weighted *N* 130)	Remaining 90 % (*N* 760, weighted *N* 1147)	*P* value for ∆
name	%FE	%TFA	%TFA	in %TFA	%FE	%TFA	%TFA	in %TFA
Pasta, rice and other cereals	6·3	1·8	3·3	<0·001	8·9	3·1	3·4	0·7
White bread	8·9	0·6	1·2	<0·001	7·7	0·9	3·2	<0·001
Wholemeal bread	2·0	0·1	0·4	<0·001	2·6	1·5	1·0	0·7
Brown, granary and wheatgerm					2·5	0·3	0·6	0·003
Other bread	2·7	0·4	1·0	<0·001	0·2	0·0	0·1	0·05
High-fibre breakfast cereals	3·0	0·9	1·0	0·9	2·2	0·2	0·2	0·5
Biscuits	2·8	12·4	6·7	<0·001	3·4	0·8	1·1	0·2
Buns, cakes, pastries & fruit pies	3·8	10·2	8·3	0·1	3·3	5·3	4·0	0·4
Puddings	1·1	1·5	1·7	0·5	0·8	3·1	1·4	0·1
Whole milk	1·7	0·9	1·6	<0·001	0·9	1·5	1·7	0·6
Semi-skimmed milk	3·1	2·2	4·4	<0·001	2·6	3·1	8·1	<0·001
Other milk and cream	0·6	0·9	1·0	0·7	0·9	6·1	2·2	0·01
Cheese	3·0	5·4	8·6	<0·001	3·4	19·2	17·6	0·4
Yoghurt, fromage frais and dairy	1·2	0·3	0·8	<0·001	1·4	0·9	2·0	0·001
Eggs and egg dishes	2·2	1·8	2·9	<0·001	2·0	1·5	2·1	0·09
Butter	1·3	2·1	4·1	<0·001	1·4	13·9	6·4	<0·001
PUFA margarine & oils	0·1	0·1	0·5	0·005	0·2	0·0	0·0	
Low-fat spread	0·4	1·5	1·8	0·6	0·3	0·0	0·2	<0·001
Other margarine fats and oils	0·6	6·3	3·7	0·3	0·8	0·0	0·2	0·2
Reduced-fat spread	1·8	14·8	5·8	<0·001	2·0	0·5	1·6	<0·001
Bacon and ham	1·6	0·2	0·4	<0·001	1·6	0·5	1·0	<0·001
Beef, veal and dishes	2·9	2·5	5·1	<0·001	2·5	5·4	8·3	0·008
Lamb and dishes	0·8	2·0	2·8	0·9	0·8	8·4	3·9	0·01
Pork and dishes	0·9	0·1	0·3	<0·001	1·0	0·3	0·7	0·003
Coated chicken	1·0	0·8	1·3	0·003	0·9	0·2	0·7	<0·001
Chicken and turkey dishes	3·9	0·6	2·1	<0·001	4·3	1·4	4·2	<0·001
Burgers and kebabs	1·2	2·0	2·5	0·3	1·0	4·2	2·7	0·5
Sausages	1·4	0·3	0·7	<0·001	1·7	1·0	2·2	<0·001
Meat pies and pastries	2·3	10·7	5·5	<0·001	1·8	0·8	1·7	0·03
Other meat and meat products	0·7	0·6	0·9	0·02	0·5	1·3	1·0	0·7
White fish coated or fried	1·2	2·0	2·1	0·9	1·2	1·5	1·8	0·6
Other white fish, shellfish	0·6	0·2	0·5	0·001	0·9	0·1	0·3	0·008
Oily fish	1·2	0·3	0·4	0·6	1·0	0·8	0·1	0·4
Vegetables not raw	3·8	1·0	1·5	0·2	4·0	2·3	1·4	0·3
Chips fried & roast potatoes	5·3	3·8	4·4	0·2	4·4	2·7	2·7	1·0
Other potatoes, potato salads	3·0	1·0	0·9	0·9	2·7	0·3	0·4	0·2
Crisps and savoury snacks	2·0	1·1	1·3	0·5	2·1	0·2	0·5	<0·001
Chocolate confectionery	2·4	3·1	3·2	0·8	2·1	1·7	2·2	0·4
Miscellaneous	3·0	1·6	3·1	<0·001	3·9	3·2	3·8	0·4
Ice cream	0·6	0·8	1·4	<0·001	0·6	0·8	1·5	0·07

%FE, percentage of food energy; %TFA, percentage of TFA intake. Percentage contributions of TFA from food types are weighted means of the individuals’ percentage contributions. Food groups contributing less than 0·5 % TFA to food energy in all TFA groups were not tabled.

### Nutritional intake of higher consumers of trans-fatty acids

Adults in the NDNS 2000/01 who consumed above the WHO TFA limit had significantly higher energy and fat intakes (absolute and as %FE), lower Na, vitamins C, D and E intakes, and lower total fruit and vegetable and vitamin C intakes per 4184 kJ (1000 kcal; Table 6). Similar differences were also found in analyses of the top 10 % of TFA consumers as a percentage of food energy, apart from Na and vitamin D, which were not significant (Table 7).

**Table 6:** Nutrient intakes of adults (aged 19–64 years) in the National Diet and Nutrition Survey (NDNS 2000/01) and Years 3 and 4 of the National Diet and Nutrition Survey Rolling Programme (NDNS RP 2010/11 and 2011/12) who consumed 1 % or more of their total energy intake from *trans*-fatty acids (TFA) compared with those who consumed less

	NDNS 2000/01					NDNS RP 2010/11 and 2011/12				
	≥1 %TE from TFA (*N* 988, weighted *N* 988)		<1 %TE from TFA (*N* 736, weighted *N* 736)			≥1 %TE from TFA (*N* 22, weighted *N* 32)		<1 %TE from TFA (*N* 826, weighted *N* 1245)		
Adults: WHO TFA limit	Mean	sd	Mean	sd	*P* value	Mean	sd	Mean	sd	*P* value
TFA (g)	3·11	1·30	1·59	0·66	<0·001	2·18	0·62	1·02	0·53	<0·001
TFA (%TE)	1·38	0·34	0·75	0·18	<0·001	1·20	0·26	0·49	0·20	<0·001
TFA (%FE)	1·43	0·34	0·81	0·20	<0·001	1·25	0·26	0·52	0·20	<0·001
Total energy (kJ/d)	8452	2582	7866	2427	<0·001	6891	1657	7673	2335	0·04
Total energy (kcal/d)	2020	617	1880	580	<0·001	1647	396	1834	558	0·04
Food energy (kJ/d)	7991	2439	7071	2117	<0·001	6643	1590	7284	2180	0·07
Food energy (kcal/d)	1910	583	1690	506	<0·001	1589	380	1741	521	0·07
Fat (%FE)	37·3	5·4	32·7	6·0	<0·001	41·0	4·9	34·2	6·1	<0·001
Saturated fat (%FE)	14·4	3·0	11·8	2·7	<0·001	18·4	3·8	12·3	3·1	<0·001
NMES (%FE)	12·5	6·2	13·0	7·5	0·2	9·4	5·3	12·0	6·4	0·05
Na (mg/4184 kJ (1000 kcal))	1520	324	1595	398	<0·001	1150	435	1295	336	0·2
Vitamin C (mg/4184 kJ (1000 kcal))	41·0	26·7	55·2	35·0	<0·001	42·4	22·6	46·3	36·2	0·5
Vitamin D (µg/4184 kJ (1000 kcal))	1·7	1·1	2·0	1·5	<0·001	1·3	0·7	1·6	1·1	0·02
Vitamin E (mg/4184 kJ (1000 kcal))	4·9	1·5	5·4	1·9	<0·001	4·5	1·4	5·5	2·3	<0·001
F&V* (g/4184 kJ (1000 kcal))	99·7	7·4	142	108	<0·001	141	89·3	168·8	112	0·2

%TE, percentage of total energy; %FE, percentage of food energy; NMES, non-milk extrinsic sugars; F&V, fruit and vegetables; TFA intake ≥1%TE is above the WHO recommended TFA limit.

* Total grams of F&V do not include pulses, baked beans and fruit juice.

**Table 7:** Nutrient intake of adults (aged 19–64 years) in the National Diet and Nutrition Survey (NDNS 2000/01) and Years 3 and 4 of the National Diet and Nutrition Survey Rolling Programme (NDNS PR 2010/11 and 2011/12) who were the top 10 % of *trans*-fatty acid consumers as a percentage of food energy compared with the remaining 90 %

	NDNS 2000/01					NDNS RP 2010/11 and 2011/12				
	Top 10 % TFA consumers as %FE (*N* 191, weighted *N* 176)		Remaining 90 % (*N* 1533, weighted *N* 1548)			Top 10 % TFA consumers as %FE (*N* 88, weighted *N* 130)		Remaining 90 % (*N* 760, weighted *N* 1147)		
Adults: top 10 % TFA consumers	Mean	sd	Mean	sd	*P* value	Mean	sd	Mean	sd	*P* value
TFA (g)	4·55	1·00	2·22	1·85	<0·001	1·93	0·59	0·95	0·46	<0·001
TFA (%TE)	1·93	0·37	1·01	0·31	<0·001	0·95	0·21	0·46	0·17	<0·001
TFA (%FE)	2·00	0·37	1·07	0·31	<0·001	1·00	0·20	0·49	0·17	<0·001
Total energy (kJ/d)	8870	3238	8117	2435	<0·001	7753	2326	7644	2326	0·7
Total energy (kcal/d)	2120	774	1940	582	<0·001	1853	556	1827	556	0·7
Food energy (kJ/d)	8494	3088	7531	2243	<0·001	7372	2151	7259	2171	0·7
Food energy (kcal/d)	2030	738	1800	536	<0·001	1762	514	1735	519	0·7
Fat (%FE)	39·7	6·0	34·8	5·9	<0·001	39·0	4·5	33·8	6·1	<0·001
Saturated fat (%FE)	15·2	3·5	13·1	3·0	<0·001	16·8	3·2	12·0	2·9	<0·001
NMES (%FE)	11·5	6·40	12·9	6·7	0·01	10·2	5·7	12·2	6·5	0·008
Na (mg/4184 kJ (1000 kcal))	1506	334	1557	361	0·09	1259	401	1294	331	0·5
Vitamin C (mg/4184 kJ (1000 kcal))	36·3	27·9	48·3	30·3	<0·001	39·5	21·9	46·9	37·1	0·02
Vitamin D (µg/4184 kJ (1000 kcal))	1·7	1·0	1·8	1·3	0·06	1·5	1·2	1·6	1·1	0·3
Vitamin E (mg/4184 kJ (1000 kcal))	4·8	1·3	5·2	1·7	0·003	4·6	1·3	5·6	2·4	<0·001
F&V* (g/4184 kJ (1000 kcal))	88·2	68·3	121	94·0	<0·001	144	72	170	115	0·008

%FE, percentage of food energy; %TE, percentage of total energy; NMES, non-milk extrinsic sugars; F&V, fruit and vegetables.

* Total grams of F&V do not include pulses, baked beans and fruit juice.

In the analyses of the recent NDNS (2010/11 and 2011/12), high TFA consumers had higher total fat, saturated fat and lower vitamin E intakes (Tables 6 and 7). Those consuming above the WHO recommendation also had lower intakes of total energy and vitamin D per 4184 kJ (1000 kcal; Table 6). The top 10 % of TFA consumers also had lower non-milk extrinsic sugars, vitamin C, and fruit and vegetable intakes.

### Analysis using predicted ‘usual’ intake distribution of trans-fatty acids

The main results above were based on mean TFA intake over the seven or four assessment days. In separate analyses using the estimated usual intake distribution, we predicted that 36·6 % in the pre-reformulation survey and none in the post-reformulation survey had TFA intake over the WHO recommendation. Having TFA intake above the WHO recommendation in the pre-reformulation survey, using this method, was associated with socio-economic disadvantage in having no qualifications or not having a degree, gross income and social class; but no significant differences were found for ethnicity (data not shown).

## Discussion

Comparison of the NDNS results representing before (2000/01) and after product reformulation (2010–2012) shows that TFA consumption in the UK has reduced substantially between these periods. In Years 3 and 4 (2010–2012) of the recent NDNS RP, which incorporates product reformulation data, only 2·5 % of adults consumed more than the current WHO recommended TFA limit (i.e. ≥1 %TE from TFA). This compares with 57 % of adults in the earlier 2000/01 NDNS. The recent survey data indicate that almost all adults are now below the UK Dietary Reference Value of 2 %FE. In addition, individuals who now have high TFA consumption tend to be consumers of products with a high natural TFA content such as butter and lamb. The range of TFA isomers derived from ruminant and industrial sources differs^(^ ^32^ ^)^. However, in studies based on replacing TFA with unsaturated fatty acids, improved lipid and lipoprotein profiles were observed for both iTFA and ruminant TFA replacement, potentially reducing CVD risk^(^ ^4^ ^)^. Prior to reformulation, high consumers generally consumed products containing iTFA. Although it appears that product reformulation has resulted in ruminant TFA becoming the dominant source of dietary TFA, the amount of ruminant products consumed on a population basis is less a cause for concern than for those containing iTFA, particularly as various positive nutrients are also gained from consuming ruminant products^(^ ^4^ ^,^ ^33^ ^)^. Additionally, there appeared to be no major changes in percentage energy from food groups over the study period that would have explained the main differences in TFA intakes. Our results are in line with the NDNS 2008–2012 report which showed no evidence that individuals from households in lower quintiles of equivalised income had higher intakes than those in the top income quintile^(^ ^18^ ^)^. Based on these results, which use average TFA values from composite product samples, it appears that voluntary product reformulation to reduce TFA consumption has been successful, with potential implications for CVD reduction.

Unadjusted analyses of the earlier 2000/01 NDNS showed that prior to reformulation, high TFA consumption was associated with lower income, lower education and having a long-term disability or illness; furthermore, having no qualifications compared with having a degree remained an independent predictor of being a top 10 % TFA consumer in the multivariate analyses. Given that higher TFA consumption is linked to higher CHD risk^(^ ^2^ ^)^, our results indicate that prior to reformulation the products’ TFA content and consumption patterns within society were likely to lead to health inequalities. Post-reformulation results from the NDNS RP (2010–2012) were less clear regarding education and income-related inequalities. The sample size was smaller in the post-reformulation survey, with lower power to find associations, and about a third more held a degree, which may have diluted any association. However, the multivariate analyses indicated that higher income groups are now more likely to be top 10 % TFA consumers than the lowest income group. High TFA consumers now tend to consume products with a high natural TFA content such as butter, lamb and cheese, which are relatively expensive products more affordable to higher income groups. Nevertheless, in the unadjusted analyses of IMD score in the post-reformulation NDNS, the top 10 % consumers were more likely to be living in the most deprived areas of England than the remaining 90 %. In addition to income, IMD incorporates other domains, including barriers to housing, services and employment. One explanation for our findings is that remote areas in the UK are generally considered deprived^(^ ^34^ ^)^ and residents may also have more traditional diets high in natural TFA.

### Strengths and limitations

A strength of the present study is the use of UK nationally representative surveys with TFA data from both before and after industrial reformulation to reduce the TFA content of food products. There were also similarities in data-gathering methods and variables produced from the NDNS data sets. However, the results are limited by self-reporting of intake, particularly in the NDNS RP (2010–2012) where individuals did not weigh their food intake and documented fewer intake days^(^ ^18^ ^)^. Weighed diet records are more accurate regarding portion size estimation than food diaries. Both have a high respondent burden so can induce significant changes in individual behaviour, but weighed diet records are more invasive and more likely to influence individual choices. For instance, they may encourage more consumption of prepared foods easy to weigh and less eating out^(^ ^35^ ^)^. There has been evidence of under-reporting in the NDNS 2000/01 in relation to energy needs^(^ ^36^ ^)^, where respondents were asked to weigh their intake over 7d. In addition, doubly labelled water feasibility studies conducted for both the earlier UK NDNS and the NDNS RP point towards under-reporting^(^ ^37^ ^,^ ^38^ ^)^. It is likely that under-reporting and inaccuracies are greater in people with lower socio-economic class or education^(^ ^39^ ^)^; such individuals who were true high consumers of TFA may have been misallocated to the lower TFA groups, potentially masking associations between social disadvantage and high TFA intake. Also disaggregation of composite dishes and allocation of the components to food groups has been more extensive in the NDNS RP than the earlier NDNS surveys; there may be an overestimation of meat and underestimation of fruit and vegetables from composite dishes in the earlier NDNS compared with the later NDNS RP^(^ ^40^ ^)^.

Weaknesses in comparing social inequalities between pre- and post-reformulation data sets include the lack of comparable IMD data or equivalised household income for the 2000/01 survey. In the post-reformulation survey there were very low numbers (*n* 22) consuming above the WHO TFA limit, indicating there may be insufficient power to find differences between TFA intake groups based on the WHO limit; therefore, we suggest that focus should lie on the results of the top 10 % analyses, especially for the recent NDNS RP 2010–2012 (*n* 88). Additionally, total energy including alcohol may have diluted the findings in relation to the WHO TFA recommendation. Lower TFA consumers drank more alcohol than high consumers pre-reformulation; higher alcohol consumers may have been more likely to be below the WHO TFA limit because TFA made up a smaller proportion of their total energy. In the UK, individuals of higher socio-economic class tend to have higher alcohol consumption^(^ ^41^ ^)^; the association between socio-economic class and TFA intake could be confounded by alcohol intake. Energy from alcohol was not excluded in the calculation to determine the groups above and below 1 %TE from TFA to remain in line with the WHO recommendation. Although energy intake from alcohol is usually a small percentage of total energy, analysis of the top 10 % in terms of food energy intake removed this potential problem.

The main analyses were based on mean intake over the seven or four assessment days; however, these short-term dietary instruments can cause overestimation of the percentage of people in the extremes, due to within-person variation, which can be pronounced for infrequently consumed foods. TFA, however, have been ubiquitous prior to reformulation in processed foods, as well as naturally occurring in meat and dairy products. Daily variability in intakes may still lead to an overestimation of the proportion above the WHO recommended limit on TFA. We used a relatively simple approach to model usual intake using the common framework method with transformed TFA intakes^(^ ^29^ ^)^, which has some limitations. As expected, this ‘usual’ intake distribution was narrower than that used for the main analysis, with 37 % instead of the original 57 % estimated to consume over the WHO TFA recommendation in the pre-reformulation survey. This group still showed associations with socio-economic disadvantage. Although the 7d data in the pre-reformulation survey could be converted to 4d data to make them more comparable^(^ ^42^ ^)^, we believe it is preferable to keep as much data as possible and more informative to present these findings for the earlier survey rather than simulated results. In any case, the population means presented should not be significantly affected by whether 4d or 7d dietary intake has been collected.

The update of TFA values in the NDNS nutrient database appears to be comprehensive for processed foods. However, the TFA values used in these NDNS surveys are an average of a small variety of popular foods and brands from large manufacturers and retailers; this average could mask important TFA differences between foods regularly purchased by different groups in society. For instance, lower income groups may consume budget foods potentially higher in TFA than the average values provided from the composite samples used in the NDNS. Lower income groups may consume products from small and medium-sized enterprises which might be less likely to supply reformulated products; such products are unlikely to be incorporated in the nutrient databanks used to calculate TFA intake in the NDNS. Further research in this area is needed to explore whether low budget or niche brand/international foods from smaller retailers have higher TFA content than the products underpinning nutrient databanks. TFA values in the food composition databanks underpinning NDNS estimates would be more representative if they were weighted by market share.

The policy context in which UK reformulation efforts occurred is important to determine the drivers of reformulation. Data from other European countries could show whether voluntary reformulation elsewhere has been effective in reducing inequalities in TFA intake. Legislated bans may be preferable where the capacity for routine monitoring of dietary intake and food composition is lower. With the Danish iTFA ban, compliance was evaluated by the government through targeted sampling and analyses of foods after the law was introduced^(^ ^13^ ^)^. A similar evaluation has not yet taken place in the UK, despite the voluntary Public Health Responsibility Deal being introduced in 2011^(^ ^10^ ^)^. The authors are not aware of any such evaluation of voluntary reformulation in other countries, and the present paper attempted to address this gap. Although voluntary, such iTFA reduction approaches are a distinct policy choice and require rigorous evaluation and monitoring, ideally styled on the ‘gold standard’ salt reduction initiatives led by the UK Food Standards Agency with government targets, reporting and monitoring^(^ ^43^ ^)^. Furthermore, caution is needed when extrapolating voluntary iTFA reduction to other policy areas, such as sugar reduction. Table sugar is considered by the industry to have functional properties that are difficult to replace, including taste profile, bulk and texture^(^ ^44^ ^)^. This makes voluntary sugar reduction potentially more challenging and is being resisted by some UK manufacturers^(^ ^45^ ^)^. Consequently, there have been calls for alternative approaches for sugar including taxes, portion size controls, restrictions on pricing promotions and mandatory limits^(^ ^46^ ^)^. Despite some technical complexity in reformulating products to remove iTFA, alternatives were available that provided the same functional attributes at minimal additional cost, while providing health benefits^(^ ^47^ ^,^ ^48^ ^)^. The shift was also fuelled by potential national or supranational legislation affecting domestic and export markets.

## Conclusion

Voluntary reformulation appeared effective in reducing the TFA content of many UK products. High TFA consumption was associated with socio-economic disadvantage pre-reformulation, but post-reformulation results were less clear regarding inequalities. Similar research should be undertaken in other European countries with voluntary reformulation as well as in countries with legislated bans. Additional research is needed to determine whether products containing high iTFA levels are still sold in the UK and whether these are more likely to be purchased by lower income groups.
